# Initial Evidence for Adaptive Selection on the NADH Subunit Two of Freshwater Dolphins by Analyses of Mitochondrial Genomes

**DOI:** 10.1371/journal.pone.0123543

**Published:** 2015-05-06

**Authors:** Susana Caballero, Sebastian Duchêne, Manuel F. Garavito, Beth Slikas, C. Scott Baker

**Affiliations:** 1 Laboratorio de Ecología Molecular de Vertebrados Acuáticos, Biological Sciences Department, Universidad de los Andes, Bogota, Colombia; 2 School of Biological Sciences, The University of Sydney, NSW, Australia; 3 Grupo de Investigaciones en Bioquímica y Biología Molecular de Parásitos, Biological Sciences Department, Universidad de los Andes, Bogota, Colombia; 4 Marine Mammal Institute and Department of Fisheries and Wildlife, Hatfield Marine Science Center, Oregon State University, Newport, Oregon, United States of America; Hokkaido University, JAPAN

## Abstract

A small number of cetaceans have adapted to an entirely freshwater environment, having colonized rivers in Asia and South America from an ancestral origin in the marine environment. This includes the ‘river dolphins’, early divergence from the odontocete lineage, and two species of true dolphins (Family Delphinidae). Successful adaptation to the freshwater environment may have required increased demands in energy involved in processes such as the mitochondrial osmotic balance. For this reason, riverine odontocetes provide a compelling natural experiment in adaptation of mammals from marine to freshwater habitats. Here we present initial evidence of positive selection in the NADH dehydrogenase subunit 2 of riverine odontocetes by analyses of full mitochondrial genomes, using tests of selection and protein structure modeling. The codon model with highest statistical support corresponds to three discrete categories for amino acid sites, those under positive, neutral, and purifying selection. With this model we found positive selection at site 297 of the NADH dehydrogenase subunit 2 (*d_N_/d_S_*>1.0,) leading to a substitution of an Ala or Val from the ancestral state of Thr. A phylogenetic reconstruction of 27 cetacean mitogenomes showed that an Ala substitution has evolved at least four times in cetaceans, once or more in the three ‘river dolphins’ (Families Pontoporidae, Lipotidae and Inidae), once in the riverine *Sotalia fluviatilis* (but not in its marine sister taxa), once in the riverine *Orcaella brevirostris* from the Mekong River (but not in its marine sister taxa) and once in two other related marine dolphins. We located the position of this amino acid substitution in an alpha-helix channel in the trans-membrane domain in both the *E*. *coli* structure and *Sotalia fluviatilis* model. In *E*. *coli* this position is located in a helix implicated in a proton translocation channel of respiratory complex 1 and may have a similar role in the NADH dehydrogenases of cetaceans.

## Introduction

Genomic evolution between species of marine and freshwater habitats has only recently started to gain attention. In euryhaline fish (fish species that migrate between saltwater and freshwater during their lifetime) a number of genes that play an important role in osmoregulation have been identified. For example, a transcriptomic study in the European eel (*Anguilla anguilla*) found 28 differentially expressed genes when the fish were maintained in freshwater or saltwater [1]. Also, a transcriptomic study in the killifish *Fundulus heteroclitus* provided evidence of the physiological plasticity in this euryaline fish and suggested the regulatory paths for the methabolic response when these fish are transferred from saltwater to freshwater environments [2]. A genomic assembly of about 20 populations of marine and freshwater three-spine stickleback fish [3] suggested that changes in the expression of regulatory loci (for example those involved in cellular signalling) are likely more predominant that those in coding sites when saltwater vs. freshwater adaptation was evaluated and that a small fraction of the genomic regions analysed showed non-synonymous substitutions between marine and freshwater fish (17% of all genomic regions analysed). Although most of these studies suggest that regulatory gene expression is a very likely mechanism involved in adaptation to freshwater environments, there is initial evidence showing some degree of amino acid structural differences found in particular proteins that may play an important role in freshwater adaptation. For instance, Whitehead [4] found evidence of fixed amino acid changes in proteins coded in the mitochondrial genome in freshwater populations of killifish when compared with marine populations.

Investigating adaptive evolution to marine and freshwater environments in mammals is not an easy task, particularly when one considers the reduced number of mammalian taxa with species represented in these two habitats. For this reason, river dolphins would be ideal candidates to conduct such investigation. Their evolutionary histories, with their ancestors evolving in marine (saltwater), and colonizing riverine (freshwater) environments make them an ideal “natural experiment” in adaptation to marine vs. freshwater habitats.

A number of cetacean species, belonging to the toothed whales or sub order Odontoceti, are found in freshwater habitats. The Amazon River dolphins (*Inia geoffrensis* and *Inia boliviensis* [5]), the very recently described Araguaian River dolphin (*Inia araguanensis*) [6], La Plata River dolphin (*Pontoporia blanvillei*), the Baiji or Yang-Tse River dolphin (*Lipotes vexilifer*), now considered extinct, and the Ganges and Indus River dolphin (*Platanista gangetica*) have been classified into four different families (Inidae, Pontoporidae, Lipotidae, and Platanistidae) [7]. Molecular studies based on analyses of mitochondrial and nuclear DNA as well as retrosposons, have provided clear evidence supporting their polyphyly and suggesting independent evolutionary trajectories in these families [7–9].

Among the family Delphinidae or ‘true dolphins’, two genera are distributed in both riverine and marine environments [8,10], arising from independent colonization events in two continents. Dolphins from the genus *Sotalia* are endemic to the Caribbean and Atlantic Coast of Central and South America and to the Amazon River and most of its tributaries [11–13] Two species have been recently accepted based on morphological, molecular, ecological and biogeographical evidence: the coastal species, Guiana dolphin (*Sotalia guianensis)* and the riverine species, the tucuxi dolphin (*Sotalia fluviatilis*) [14].The second genera, *Orcaella*, has two valid species. The Irrawaddy dolphins (*Orcaella brevirostris*) are distributed in coastal and riverine habitats of Asia, including coastal and riverine environments of India, Indonesia, Malaysia, Vietnam and Cambodia [15]. A fully riverine population of Irrawaddy dolphins is found in the Mekong River in Cambodia [16]. These animals spend their complete lifespan in freshwater. The snubfin dolphin (*Orcaella heinsohni*) is a separate species and is found in coastal environments of eastern and northern Australia [17].

Colonization of riverine habitats has occurred independently and at different times in these groups. The ancestor of *Inia* and *Pontoporia* may have colonized brackish water environments in continental seas in the middle Miocene [7]. In contrast, the genus *Sotalia* originated in the Atlantic, colonizing the Amazonian habitat around 2–2.5 MYA (Million Years Ago) [11,14,18] during the Plio-pleistocene [14].

Considering the very different environments where riverine vs. marine odontocetes are found, one would also expect ecological adaptation, directed by divergent natural selection, to have shaped the evolutionary history of these groups. Possible environmental pressures that could have influenced ecological adaptation would be differences in salinity found between, for example, the main course of the Amazon river and its mouth [19] or between coastal areas of Cambodia and the Mekong River [15].

Experimental studies on fish exposed to different salinities suggested that differences in the net cost of swimming and energy required for osmorregulation was higher when the fish were maintained in freshwater, compared to the net cost of swimming and osmoregulation in seawater [20]. Recently, Whitehead et al. [4] suggested increased energetic and metabolic requirements and adjustment in killifish in initial phases of acclimation to freshwater. Interestingly, transcriptomics studies of osmorregulation in European eels [1,21], have revealed a number of genes that appear to have an important function in fish that undertake migrations between freshwater and saltwater during their lifespan [21]. Although several of these genes were located in the nuclear genome, a few genes were coded in the mitochondrial DNA, which would also be a likely target for selection due to its functional role in energy metabolism. For example, the NADH dehydrogenase, which is coded by both nuclear and mitochondrial genomes, appeared to be upregulated in kidney cells when European eels were maintained in freshwater and it was down-regulated after two days once the fish was transferred to saltwater [1]. Therefore, these results suggested that this protein complex may have an important role in osmoregulatory processes, by increasing respiratory activity and energy production in the kidney.

Considering the high number of genes found in the mtDNA that code for proteins involved in oxidative phosphorylation, one could hypothesize that selective changes in some of these genes may influence the metabolic performance of particular organisms [22], as was suggested for the killifish [4]. On the other hand, due to the functional importance of mitochondrial genes, it has been suggested that purifying selection would be the dominant force in their evolution [22], preventing fixation of detrimental mutations. However, episodic positive selection may occur if selective pressures shift, possibly by changes in the environment [22]. Recently, evidence of adaptive evolution of the mtDNA genome in mammals has been detected [23,24], suggesting that it may have facilitated the radiation and successful diversification of mammals to very different environments (aquatic vs. terrestrial, cold vs. warm, etc) [23]. In these studies, evidence for positive selection was found on a number of mitochondrial genes in various mammalian taxa, including, ND1, ND2, ND4 and ND5, which code for subunits of the NADH dehydrogenase. The authors suggest that such positively selected changes in these genes may be related to their possible role as proton pumping devices and could be related to energy demands in species such as shrews, moles, hedgehogs but also African elephants. Also, positive selection was detected in 11 out of 13 mitochondrial genes in two subterranean South American rodent lineages, the tuco-tuco (*Ctenomys*) and the coruro (*Spalacopus*) [22]. The authors of this study suggested that weak positive selection, against a background of purifying selection, appear to have resulted in convergent adaptive evolution in the mitogenomes of these rodents, allowing them to successfully colonize subterranean habitats, characterized by low oxygen availability. In cetaceans, positive selection in the Cytochrome *b* gene of the killer whales (*Orcinus orca*) from Antarctica, may be related to the need for higher metabolic rates required for survival at low temperatures [25]

We hypothesize that selection on mitochondrial of mitochondrially-encoded proteins was necessary for the adaptation of cetaceans to the freshwater environment because of increased energetic and metabolic needs accompanying osmoregulation in freshwater. Body fluids of cetaceans found in marine environments have a lower ionic content than the seawater environment [26] and they obtain freshwater mainly from their food. Nevertheless, their kidneys are reniculated, which helps to concentrate urine efficiently [26]. Reniculated kidneys have also been described in freshwater cetaceans which has revealed no major differences in renal function [27]. In addition, one study found that the kidney of the freshwater Ganges river dolphin was only 62% of the size of a marine dolphin of the same size [28]. Unfortunately, no additional information is available to date that could explain how freshwater cetaceans efficiently eliminate excess water while retaining solutes.

We hypothesize that such mechanism would prevent kidney damage due to oxidative stress, but may result in increased metabolic needs [29].

We also hypothesize that convergence in adaptation in these proteins was likely to occur given the independent colonization events by different cetacean groups.

In order to test this, we analyzed full coding sequences from the mitochondrial genomes of 29 odontocete species for evidence of positive diversifying selection. Additionally, since genes coding for the NADH dehydrogenases appear to be related to osmoregulation in fish, we wanted to test if this protein had fixed aminoacid changes among freshwater cetaceans that could be indicative of adaptive convergence.

## Materials and Methods

### Sampling and DNA extraction

Skin samples (less than 1 cm^3^) were obtained from the tail of dolphins found stranded dead or drowned in fishing nets including one Guiana dolphin (*Sotalia guianensis*) from Santa Marta, Colombian Caribbean, three tucuxi dolphins (*Sotalia fluviatilis*) from Tarapacá and Puerto Nariño in the Colombian Amazon, one Mekong Irrawaddy dolphin (*Orcaella brevirostris*) from Kratié, Cambodia, one Atlantic spotted dolphin (*Stenella frontalis*) and one spinner dolphin (*Stenella logirostris*) from Puerto Rico and one Amazon River dolphin (*Inia geoffrensis*) from Arauca, Colombian Orinoco. Samples were obtained in the Colombian Amazon and Colombian Caribbean under authorization granted by Ministerio del Medio Ambiente y Desarrollo Territorial (Contrato de Acceso a Recursos Genéticos No. 001 granted to S. Caballero) and collected by employees from the regional environmental authorities (Corporaciones Autónomas Regionales) and Fundación Omacha. Samples from the Mekong Irrawady dolphin were obtained by WWF Cambodia under authorization granted from the Cambodian Government as part of the Mekong Irrawaddy dolphin Recovery Plan and imported to Colombia complying with all CITES documentation (No. KH0669 and E-04406/09). These samples were collected by the veterinarians working in the Mekong Irrawaddy dolphin Recovery Plan. The *Stenella frontalis* and *Stenella longirostris* samples were obtained by personnel of the Caribbean Stranding Network and import and export of samples was carried out in the US under Marine Mammal Protection Act permits and CITES permits issued to the National Marine Fisheries Service (NMFS). No animals were killed for use in this study. DNA was extracted using the tissue extraction kit from QIAGEN.

### Mitochondrial genome amplification and sequencing

Mitochondrial genomes were obtained by amplification of six fragments. Each of these fragments where flanked by a set of particular primers (Table 1). Primer design was achieved by comparisons with previously published mitochondrial genomes from other cetaceans, such as sperm whales [30] and by comparisons with primers previously designed by T. Mclenachan at the Alan Wilson Centre, Massey University, Albany, New Zealand. Table 1 shows primer pairs designed for this study and their annealing temperatures. All PCR reactions were performed using 2u/μl of Phusion High Fidelity DNA Polymerase (Biolabs), HF Buffer (5X), BSA, 10 μM of each primer, DMSO and 20mM dNTPs. The basic PCR temperature profile was as follows: an initial denaturation step at 98°C for 30s, followed by 35 cycles of 8s at 98°C, annealing at 64°C for 30s, extension at 72°C for 1m 15s and a final extension for 20m at 72°C. For primer pair DelHS13660F-Del12sRNAR, a slightly different amplification protocol was followed, using touchdown: and initial denaturation step at 98°C for 30s, followed by 3 cycles of 8s at 98°C, annealing at 64°C for 30s, extension at 72°C for 1m 15s, followed by 3 cycles of 8s at 98°C, annealing at 63°C for 30s, extension at 72°C for 1m 15s, followed by 30 cycles of 8s at 98°C, annealing at 62°C for 30s, extension at 72°C for 1m 15s, and a final extension for 10m at 72°C.

**Table 1:** Primers used for amplification of mitochondrial genomes and the ND2 gene of cetacean species included in this study.

Fragment	Forward primer	Sequence (5´-3´)	Reverse primer	Sequence (5´-3´)	Annealing T (°C)
1	1.4 UPF	AATCCAGGTCGGTTTCTATCT	DelNDR	CAATTGATGAGTAGGCTATAATTTTC	64
2	Pma6800CO1F	GAGAAGCMTTYRCATCCAAACG	DelHDND4R	GGGGTCAGAGAAGAATATTAAAGA	62
3	Mys10000ND4LF	CGATCCCACCTAATRTCCGCA	Mys13000ND5R	GCTCAGGCGTTGGTATAAGA	64
4	DelHS13660F	GCCTCAACCAACCACACCTAG	Del12sRNAR	GTGCTTGATGCCCGCTCCTTTT	TD (64,63, 62)
5	DelM13tpheF	AAAGCAAGACACTGAAAAATGCT	DelHD3106R	TAGACAGTTAGGCTTGATATTGT	62.5
6	DelND2F	GAAAATTATAGCCTACTCATCAATTG	Pma6916tSerR	GTTCGAKTCCTTCCTTTCTT	64
7 (ND2 amplification)	LP5100R	AGGCTTTTGAAGGCCTTTGGTCT	HS4823tRNA-metF	CCCATACCCCGGAAATGTTG	55
8 (ND2 sequencing)	BatL4235	TTTCACTTTTGARTACCAGAAGTT	BatH4461	TGGGCRATTGATGAGTATGC	55

Primers designed for this study are shown in bold. TD refers to touchdown PCR.

Successfully amplified fragments for each sample were combined and cleaned using the QIAquick PCR purification kit (QIAGEN) and the cleaned PCR products were quantified using a nanodrop. Libraries were prepared for each sample and sequenced following the protocols for the 454 Roche GS Junior Titanium Series Sequencer.

Given the apparent importance of ND2 in osmorregulatory function in fish, we wanted to investigate if some sites in this gene were under positive selection and if they were fixed among freshwater species. Therefore, additional ND2 gene sequences (1044 bps) were obtained using published amplification protocols by Caballero et al. [14] and traditional Sanger sequencing on an ABI 3100 at Universidad de los Andes (Bogotá, Colombia). This sample set included an additional ten *Sotalia fluviatilis* samples, 14 *Sotalia guianensis* samples, eight Mekong River *Orcaella brevirostris* samples and four *Inia geoffrensis* samples from a wider geographic area, including two *Inia geoffrensis humboldtiana* (subspecies from the Orinoco) and two *Inia geoffrensis geoffrensis* (subspecies from the Amazon).

### Sequence assembly and annotation

Contigs for all mitogenome sequences were assembled using Velvet v1.2.1 [31] and ordered into scaffolds using Mauve v2.3.1 [32]. The annotations of individual genes was performed in Genious v5.4 [33] using reference mitogenomes available in GenBank. We used the complete mitogenome of *Orcinus orca* (ACCN: GU187211) for all delphinids, and that of *Inia geoffrensis* (ACCN: AJ554059) for our *Inia* samples. Genome annotations were imported from the reference sequences and reading frames were visually inspected for frame-shifts or anomalies in the new sequences.

Sequence coverage was highly variable among samples, with some regions having low sequence coverage. Therefore nucleotides for these regions are labelled as N in the final sequences and were submitted as partial mitogenomes to GenBank with accession numbers KM893421 to KM893428.

### Selective constraints and phylogenetic analyses

We tested for selective constraints in all coding regions, except for *ND6* [34]. To allow for broad hypothesis testing, we included previously published mitogenomes from other members of Delphinidae [35–37] and Inidae [38] available in GenBank. The resulting dataset contained 36 sequences for the 12 genes (see S1 Table for mitogenome accession numbers used for comparison).

We aligned the protein coding regions in CLUSTAL W2 [39] of the mitogenomes, with the exception of *ND6*, to produce a concatenated data set. Then, we estimated a Maximum Likelihood phylogenetic tree in the Phangorn R package [40]. The substitution model was GTR+G+Γ, according to the Bayesian information criterion. To assess node support, we conducted 1000 boostrap replicates. This tree topology was used for all subsequent analyses. To investigate the selective constraints we extracted individual genes and aligned them in CLUSTAL W2. Since some genes, such as *ND1* and *COII* were missing large portions for some of the sequences, we either excluded them altogether or ignored the sites with missing nucleotides in subsequent analyses.

We tested the Nielsen and Yang [41] codon models implemented in HyPhy v2.1 [42] for each coding region. We chose this approach over other methods, because it includes a very comprehensive set of candidate codon models, including those available in other popular packages, such as PAML[43], Importantly, these models have sufficient statistical power to detect differences in selective constraints, even for intra-population data, or sequences with low overall variation (for reviews on these methods see [44] and [45]). The models tested under this framework use a maximum likelihood approach, where the global rate of nonsynonymous and synonymous sites (*d*_*N*_ */d*_*S*_) is sampled from an array of distributions and a posterior probability is assigned to each site for *d*_*N*_ */d*_*S*_>1, indicating positive selection. We calculated the Akaike Information Criterion (AIC) for the 15 models available in the software (S2 Table), and selected the best-fitting models accordingly. For all discrete models we selected three categories of *d*_*N*_ */d*_*S*_. The model with the lowest AIC score was selected to make inferences of positively selected sites. In one additional analysis, we used a dataset that included additionally sequenced *ND2* for which the complete mitogenomes were not available (Table 2). We conducted this additional analysis on this gene because of its possible role in adaptation to freshwater habitats, in order to increase the geographic coverage and sample size of our study.

**Table 2:** Selection models tested.

Gene	Best Model	Number of parameters	Global dN/dS	variance dN/dS	Proportion of sites with dN/dS>1 (P>0.5)	dN/dS positive selection threshold	Notes
COI	MODEL 14 (Gamma mod Beta)	4	0,49	0,16	0,11	1,48	Large missing data
COII	MODEL 15 (Beta & 1)	4	0,24	0,05	0,00	1,00	No positive selection in the model
COIII	MODEL 3 (Discrete)	5	0,32	0,13	0,05	1,80	Large missing data
ND1	MODEL 2 (Selection)	4	0,72	0,67	0,14	2,57	No fixed differences between marine and riverine taxa
ND2	MODEL 3 (Discrete)	5	0,79	0,55	0,19	2,23	Positively selected sites detected
ND3	MODEL 2 (Selection)	4	0,76	0,86	0,01	8,07	Large missing data
ND4	MODEL 4 (Freqs)	4	0,58	0,49	0,06	3,00	Large missing data
ND4L	MODEL 8 (Beta & w)	4	0,22	0,09	0,10	1,00	No positive selection in the model
ND5	MODEL 14 (Gamma mod Beta)	4	0,59	0,40	0,18	1,80	Large missing data
atp6	MODEL 8 (Beta & w)	4	0,26	0,09	0,13	1,00	No positive selection in the model
atp8	MODEL 15 (Beta & 1)	4	0,18	0,08	0,10	1,00	No positive selection in the model
cytB	MODEL 2 (Selection)	4	0,70	0,36	0,10	2,24	No fixed differences between marine and riverine taxa
ND2 with additional sequences	MODEL 10 (Beta & (Gamma+1))	4	0,67	0,13	0,19	1,07	Positively selected sites detected

### Protein modelling and structural analysis

Inferred amino acid sequences were aligned using the ClustalW2 server (European Bioinformatics institute). The 3D homology models of ND2 of *S*. *guianensis* and *S*. *fluviatilis* were generated by the SWISS-MODEL server [46] (Swiss institute of bioinformatics) using subunit D of the protein structure *NuoN* in *Escherichia coli* [47] as a template (PDB: 3RKO). Posterior optimization, molecular graphics and analyses were performed with the UCSF Chimera package [48] and the SWISS-Pdb viewer v4.1[49]. Both, structure and models were minimized using the default Chimera 100 steps of conjugated minimization steps.

## Results

### New mitogenomes generated

**Fig 1:**
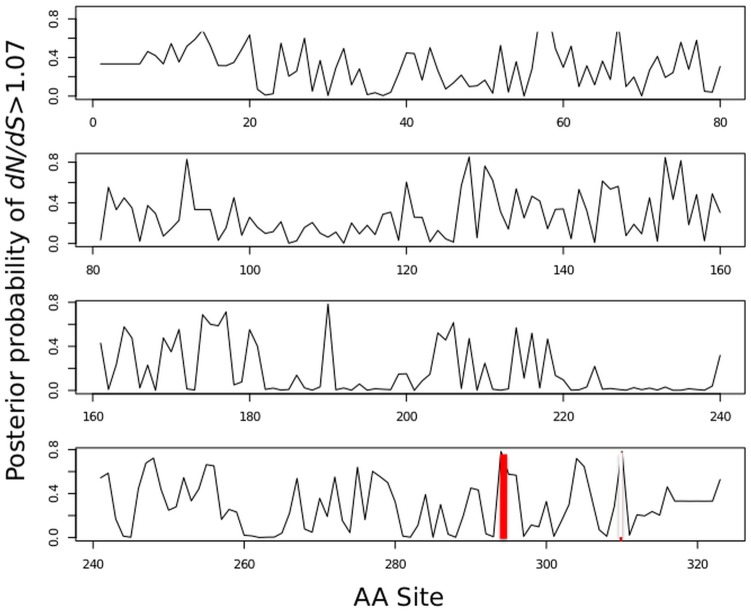
Phylogenetic tree estimated from mitochondrial genome sequences (n = 29) using Maximum likelihood. Statistical support for all nodes is 100%, according to 1000 bootstrap.

Eight new mitochondrial genomes were sequenced, assembled and annotated in this study for six odontocete species, including one Guiana dolphin (*Sotalia guianensis*), three Tucuxi dolphins (*Sotalia fluviatilis*), one Mekong Irrawaddy dolphin (*Orcaella brevirostris*), one Atlantic Spotted dolphin (*Stenella frontalis*), one Spinner dolphin (*Stenella logirostris*) and one Amazon River dolphin (*Inia geoffrensis*). These new assembled mitogenomes, as well as some previously published ones (n = 21), were analysed individually in the 12 different protein coding regions for variations between freshwater and seawater species. We excluded *ND6* [34] from the analysis because it is located in the heavy strand of the molecule and it displays patterns of nucleotide substitution that are difficult to model with standard substitution models. The Maximum likelihood tree was congruent with previous studies with nuclear and mitogenomic data sets and yielded 100% bootstrap support for all nodes (Fig 1).

### Codon models and tests for selection in the mitogenome

The codon models selected according to the AIC indicated that a proportion of sites were under positive selection in the 8 genes: *COI*, *COIII*, *ND1*, *ND2*, *ND3*, *ND4*, *ND5*, and *Cytb* (Table 2). However, mapping the substitutions showed that only *ND1*, *ND2*, and *Cytb*, have fixed differences between marine and riverine species, across the available mitogenomes. We examined positively selected sites, as suggested by the codon model with highest statistical support to detect fixed mutations in riverine taxa (Table 2).

**Fig 2:**
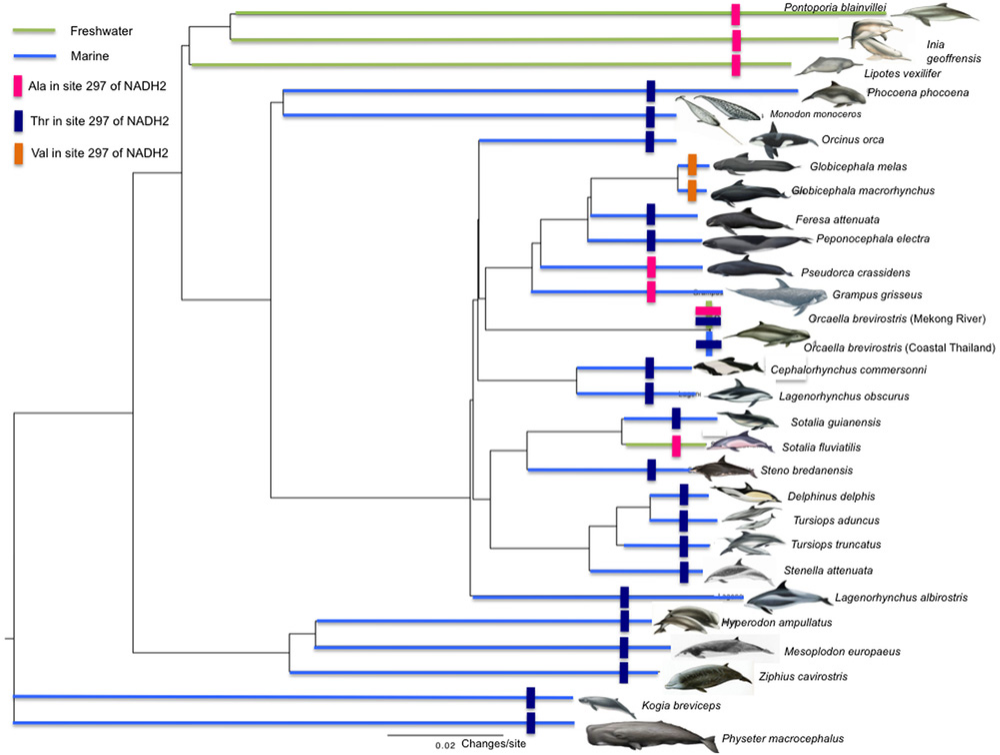
Posterior probability per site for *d*_*N*_ */d*_*S*_ >1 (in practice *d*_*N*_ */d*_*S*_ = 2.24 given the best-fitting codon model) across the *ND2* gene. The red point corresponds to site 297

We used the best fitting models for *ND1*, *ND2* and *Cytb* to separate the substituted sites into three discrete *d*_*N*_ */d*_*S*_ categories: 0.21, 0.74, and 1.07, the latter indicating the threshold *d*_*N*_ */d*_*S*_>1 which classifies a site as under positive selection, which is similar to the M3 model in PAML[50]. Only the *ND2* gene was identified as a candidate under this criterion. From the six codons sites that show non-synonymous substitutions (T8I, I139V, F159L, T297A and L343F) between the taxa of fresh and salt water, only codon site 297 had a high posterior probability of assignment to the third category, suggestive of positive selection (Probability of assignment of = 0.78) (Fig 2). The amino acid for this site was a Threonine (Thr) in 22 marine species (Fig 2), Valine (Val) in two marine species (*Globicephala melas* and *Globicephala macrorhynchus*) and Alanine (Ala) in five freshwater species (*Inia geoffrensis*, *Lipotes vexilifer*, *Pontoporia blainvillei*, *Sotalia fluviatilis* and *Orcaella brevirostris* from the Mekong) and in two marine species (*Grampus griseus*, *Pseudorca crassidens)* (Fig 1). Notably, Ala was found in all three of the ancient ‘river dolphin’ lineages and in both of the freshwater species of Delphinidea. This amino acid substitution involves general changes in chemical properties such as the molecular weight (Ala having less than half of the molar weight of Thr) and polarity (Ala non-polar and Thr being polar). More specifically this modification has an effect on the hydrophobicity index of the site, switching from a polar -0.7 kcal mol^-1^ of Thr to a hydrophobic 1,8 kcal mol^-1^ of Ala. In *G*. *melas* and *G*. *macrorynchus*, Val was found at this site, which is structurally similar to Thr, hydrophobic and non-polar.

### Additional ND2 sequence analyses

**Fig 3:**
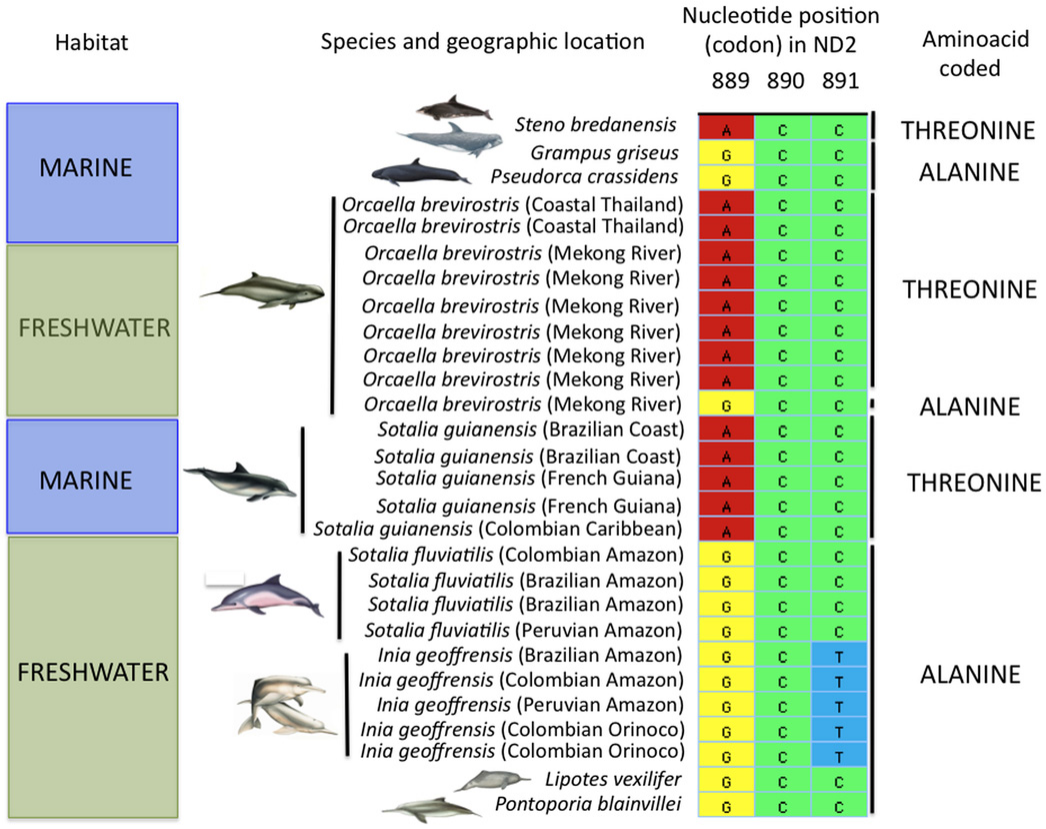
Diagram describing nucleotide substitution and codons coding for the aminoacid at site 297 of the ND2 in freshwater and marine odontocete species.

Given the phylogenetic evidence for adaptation to freshwater habitats at the *ND2* codon site 297, we included additional sequences of this gene from samples of *Sotalia fluviatilis*, *Sotalia guianensis*, *Orcaella brevirostris* and *Inia geoffrensis* (Fig 3) representing a wider geographic area of each species distribution. We found that all *Sotalia fluviatilis* shared the codon GCC for Ala, rather than the codon ACC for Thr, found in *Sotalia guianensis* and most other marine cetaceans. For *Inia geoffrensis*, we found all samples shared the codon GCT for Ala. For *Orcaella brevirostris* from the Mekong River, we found that this substitution was not fixed. We found the codon GCC for Ala in one of the samples (the reference sample used in the mitogenome), but the codon ACC for Thr in six other samples. The codon GCC for Ala was shared In the river dolphins *Lipotes vexilifer* and *Pontoporia blainvillei*, as well as in the marine delphinids *Grampus griseus* and *Pseudorca crassidens*.

### Structural analyses of ND2 and position 297

#### Complex I and likely role of subunit ND2

The ND2 subunit is part of the membrane domain of complex I (EC 1.6.5.3) also referred to as NADH dehydrogenase, the first and largest enzyme of the respiratory chain (S2 Fig). Complex I has eight hydrophilic subdomains which couple the electron transfer between NADH and ubiquinone, while the additional hydrophobic subdomain translocates the protons across the membrane [51]. The α-helical membrane-bound hydrophobic domains of complex I contain seven core subunits that are mitochondrially-encoded in eukaryotes (ND1-ND6 and ND4L). The three largest subunits ND5 (homolog of NuoL in *E*. *coli*), ND4 (NuoM), and ND2 (NuoN) are thought to be the proton pumps of Complex I [47]. These three subunits, likely participate in proton translocation as they are homologous to each other and to a particular class of H^+^/Na^+^ antiporters [52]. However, the exact mechanism that couples the redox and proton-transfer reactions is still debated, between a ‘direct’ (redox-driven) and ‘indirect’ (conformation-driven) model [51,53].

Based on highly conserved amino acids and a structural comparison with *E*. *coli subunit NuoN*, the dolphin ND2 antiporter subunit likely transports a hydrogen ion across inner-mitochondrial membrane by coordinating two half-channel reactions (conformation-driven model). A first conformational change moves a glutamic acid in TMα2 away from a protonated lysine in TMα4a, forcing its proton into the link between the two half-channels. Then, in a second local conformational change, a second deprotonated lysine in TMα9b receives the proton. At this point, the glutamic acid in TMα2 moves back and the lysine in TMα4a is protonated again from the mitochondrial matrix. This movement reloads the pump and the lysine in TMα9b ejects the proton into the intra-membranous space, providing a proton flux force that adds to the electrochemical gradient used for the synthesis of ATP in the oxidative phosphorylation [51,53].

### *Sotalia sp*. ND2 model analysis

**Fig 4:**
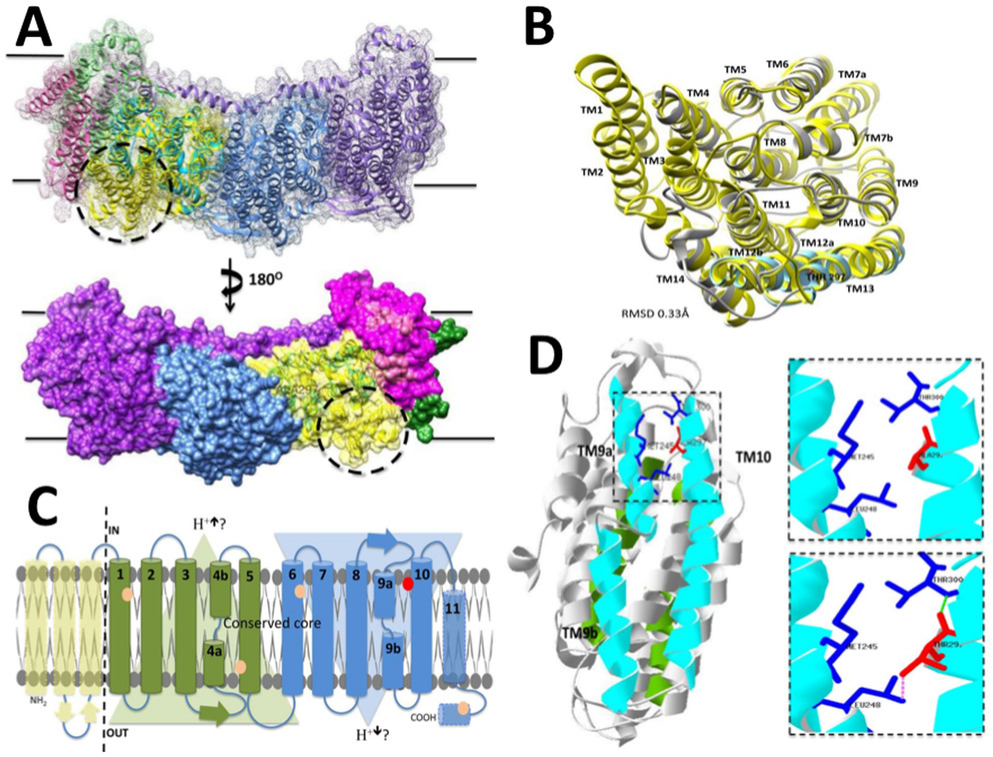
Panel A. Molecular surface comparison of the ND2 truncation in dolphins over the *E*. *coli* respiratory complex I membrane domains. Molecular surface and secondary structure of the *E*. *coli* complex I membrane domains structures (PDB: 3RKO) superimposed to the ND2 model of *S*. *guianensis*. *E*. *coli* subunit surfaces are colored as follows: NuoL, purple; NuoM, blue; NuoN, yellow; NuoA, pink; NuoJ, green and NuoK, fuchsia. *S*. *fluviatilis* ND2 subunit colored in Cyan and alanine position 297 labeled. Dashed circle highlights the TM helixes of *E*. *coli* not present in *S*. *guianensis*. Panel B. Superimposed structures of the predicted ND2 model from *S*. *fluviatilis* and the NuoN subunit in *E*. *coli*. Figure shows the structural overlap between template (PDB: 3RKO) in yellow and the model in grey. Transmembrane helixes of *E*.*coli* are labelled TM1-14. *S*. *fluviatilis* TM9a, TM9b and TM10 are colour cyan and in TM10 Threonine at position 297 is highlighted in red. Panel C. Topology diagram of the antiporter-like subunits of ND2 from *S*. *fluviatilis* and NuoN subunit in *E*. *coli*. Two inverted repeats over the conserved core of ND2 in the model, represent the internal structural symmetry and are shown in green and blue. In transparent yellow, representation of N-terminal part of *E*. *Coli* not present in *Sotalia sp*. In transparent green the C-terminal of *E*. *Coli*, present but not well conserved in the *Sotalia sp*. models. Position 297 is indicated as red dot. Possible function as sodium proton antiporter displayed as Na+/ H^+^?. Panel D. Structural analysis of position 297 substitution over the ND2 model between riverine *S*. *fluviatilis* and marine S. guianensis dolphins. Predicted structure of the subunit ND2 of *Sotalia sp*, in grey; Transmembrane helixes TM4a, TM4b and TM5, in green; and TM9a, TM9b and TM10 in cyan. Position 297 is highlighted in red and the amino acids in close proximity (Met 245, Leu248 and Thr300) in blue. Left side: Over the model structure the dashed box highlights key residues in the space of close proximity to position 297. Upper-right side: *S*. *fluviatilis* model showing Ala in position 297 in red; Lower-right side: S. guianensis model showing Thr in position 297 in red; potential hydrogen bond shown as a dashed green line and possible interaction of the rotamer shown as a dash pink line.

To explore the possible structural and functional effects that the positively selected site substitution has on the *Sotalia* complex I, a protein model of the ND2 subunit was generated for the riverine *S*. *fluviatilis* and the marine *S*. *guianensis* using the *E*. *coli* structure as a template. When the template and *Sotalia* ND2 models where superimposed, the calculated RMSD (root squared mean deviation) was 0.33Å and in the optimized models 0.19Å, suggesting a good degree of global structural similarity in the conserved core with only 1.3% of the amino acids in disallowed regions (Fig 4B).

Structurally, ND2, ND4 and ND5, displayed a main conserved secondary arrangement that consisted of 14 TMα and exhibited internal symmetry of the subunit core of ten TMα (helix 1–5 and 6–10 in a face-to-back position, see Fig 4C [21,53]). The *Sotalia sp*. ND2, as that of other higher metazoans, displayed a truncated subunit when compared to its bacterial counterparts, missing the first part of the N-terminus, involving helixes TMα1, TMα2 and TMα3 (S1 Fig). This truncation in the ND2 subunit represents a loss of part of the molecular surface of the membrane domain of respiratory complex I in dolphins when compared to *E*. *coli* (Fig 4A). Whether or not this missing surface can be replaced in dolphins by the attachment of a different nuclear-encoded protein, as could be the case in human ND2, still needs to be determined. However, no supernumerary subunits have been found attached to the mammalian mitochondrial ND2 structure obtained for *Bos Taurus* [54]. Also, Blast searches using the N-terminal regions (TMα1, TMα2 and TMα3) failed to identify any candidate proteins using the bilaterian data set of NCBI [52].

A multiple sequence alignment in conjunction with the prediction of transmembrane helices (S1 Fig) allowed us to identify the structural locations of the six sites that displayed non-synonymous substitution between riverine and marine species. Since none of the six substitutions were located directly over the TMα4a and TMα9b, they are not likely to be in direct contact with the lysine residues (present in *Sotalia sp*) key in the conformation-driven model [53]. The schematic representation of the *Sotalia sp*. ND2 and the substitutions sites can be seen in Fig 4C; in which site 297 seems to be located internally as part of a second antiporter-like symmetric subunit facing the mitochondrial matrix side. The positively selected substitution at site 297 from Thr in the marine to Ala in riverine dolphins is found near the end of TMα10, which is adjacent to TMα9b. From our models, we suggest that this substitution could have a more indirect effect by being able to provide a less restrictive environment between TMα10 and TMα9a. This could facilitate the conformational movement of TMα9b, avoiding a possible hydrogen bond interaction with Thr300 and limiting the interaction with Leu248 (Fig 4D). However, since ND2 displays structural similarity to the transmembrane MrpD sodium-proton antiporters [55], it is possible that a more complex mechanisms of translocation involving the proton movement and maybe also movement of Na^+^ could be taking place (S2 Fig).

## Discussion

Here we describe and provide initial evidence of positive selection in the NADH dehydrogenase subunit 2 for distantly related species of riverine dolphins by means of full mitochondrial genome sequencing, phylogenetic analyses, tests of selection and protein structure modeling.

As shown in Table 2, the main amino acid candidate for adaptive evolution in *Sotalia fluviatilis* is residue 297 in the *ND2* gene, perhaps having some functional importance in the speciation processes in different habitats, since it is common to all *Sotalia fluviatilis*, one Mekong River dolphin *Orcaella brevirostris*, all *Inia geoffrensis*, one *Lipotes vexilifer* and one *Pontoporia blainvillei*, and it is uncommon in other marine delphinids (found only in *Grampus griseus* and *Pseudorca crassidens*), and is absent in all *Sotalia guianensis* and in two coastal *Orcaella brevirostris* and six Mekong River *Orcaella brevirostris*, with a relatively high (>1.07) *d*_*N*_ */d*_*S*_ ratio among the Delphinidae, suggesting than this substitution is convergent rather than an ancestral polymorphism. However, the fact that two marine species also share this substitution may indicate that it is not detrimental to habitat use in marine environments. We hypothesize that this substitution could provide a “preadvantage” when colonizing freshwater habitats.

Hypothesizing that this site is relevant for adaptation to freshwater systems, we suggest convergence mediated by selection at this site, given that the same residue is found in all riverine species in our study. *S*. *fluviatilis* and *Inia geoffrensis* are sympatric in the Amazon River system and are under similar ecological conditions.

Based on molecular and paleontological evidence, it is believed that *Inia geoffrensis* and *Sotalia fluviatilis* last shared a common ancestor around 11.5 MYA [56] so incursion and adaptation into freshwater habitats took place at different time frames in each species. Random convergence and fixation of this substitution seems unlikely given that Ala in the 297 position is only present in these two species, in *Lipotes vexilifer* and *Pontoporia blainvillei*, and also in one riverine *Orcaella brevirostris* as well as in two marine delphinids. We suggest that this site is a candidate for early adaptation into freshwater given the recent divergence of *Sotalia fluviatilis* from its marine sister species and its presence in other freshwater dolphins. The finding of both Thr and Ala at this site in *Orcaella brevirostris* from the Mekong River, is difficult to explain. We suggest that this may be due to a much more recent colonization and establishment of fully freshwater populations of this species in some Asian rivers with not enough evolutionary time for this site to get fixed in this population. However, to date, no information is available regarding the possible date of colonization of Asian rivers by this species.

We hypothesize that convergence of this amino acid substitution in ND2 in riverine odontocetes may be associated with increased energy requirements for life in the freshwater environment as has been shown in some euryhaline fish [2,20]. Therefore, these results support the idea that this protein may have an important role in osmoregulatory processes, by increasing respiratory activity and energy production in the kidney [26,29].

The L-shaped NADH dehydrogenase plays a central role in energy transduction and consists of a peripheral and a membrane arm [23]. It catalyzses the oxidation of NADH, the reduction of quinone and translocates cations across the membrane. This contributes to the generation of the transmembrane electrochemical potential which is used for ATP synthesis and solute transport. The mammalian complex I of *Bos taurus* contains 44 different nuclear and mitochondrial encoded subunits [23,57]. Some of the 14 core subunits are encoded in the nuclear DNA genome (subunits in the “peripheral arm” [23,53]), and seven subunits are encoded in the mitochondrial DNA [23,58] (“membrane arm”). The nucleotide substitution rate in mitochondrial genes is often faster than rates of many nuclear genes, therefore is not surprising to find different mutation in this subunits.

The membrane arm includes subunits *ND2*, *ND4* and *ND5* which also connect by a piston arm this translocating machinery. They are suggested to be proton-pumping devices which are related to Na+/H+ antiporters of the Mrp family [23]. It has long been established that complex I translocates H^+^, but there is growing evidence that indicates that Na^+^ could be directly involved in the catalytic mechanism of Complex I [59]. It is thus possible, that the antiporter-like pumps in mammals not merely provide proton translocation but may still retain a true antiporter function capable to translocate Na^+^ as well as H^+^ [60,61]. For mammals and other vertebrates, mutations in these subunits may interfere with the efficiency of the proton-pumping process and could hinder or improve the proton translocation [23,62]

Taking this into consideration, we suggest that in riverine odontocetes that have adapted to a fully freshwater environment, increased efficiency in proton flow would improve efficiency of ATP production. This possible increase in proton flow could be achieved by amino acid changes such as the one detected in position 297 between two riverine delphinids and their marine sister species, which is also convergent for three other species of river dolphins. We suggest that this change could facilitate proton translocation in NADH Complex I in riverine odontocetes, allowing a less restrictive space for the movement of TMα9b involved in H^+^ translocation. However, another possibility could be that position 297 may be also involved in Na^+^ translocation. In our natural experiment, the NaCl environment markedly influences the osmotic gradient encountered by the mitochondria of marine and freshwater species. It seems reasonable, that a Thr in position 297 compared to an Ala could allow a more restrictive space for the flow of Na^+^ into the mitochondrial matrix. It could be important in order to maintain the balance between the internal and external compartment opposing the net osmotic gradient. Whether this substitution provides a true conformational movement advantage to the riverine dolphins, affecting the H^+^ or Na^+^ transport activity, or plays a key role in the adaptive context of riverine vs marine taxa in ND2, requires further biochemical evidence.

Additional analyses of whole mitogenomes and particularly the ND2 gene of riverine vs. marine manatees, or freshwater vs. marine seals (such as Baikal Lake seals *Pusa sibirica*) would provide an interesting comparison to support our findings regarding the role of this gene in freshwater adaptation.

## Supporting Information

S1 Fig Structural alignment of the NADH dehydrogenase subunit 2 of freshwater and marine dolphins using *E*. *coli* membrane subunit NuoN. Alignment of the S. guianensis and S. fluviatilis predicted amino acid sequence with *E*. *coli* NuoN (PDB: 3RKO:D). Secondary structural representation of the predicted dolphin NADH dehydrogenase (EC 1.6.99.3) subunit 2 motifs from the PDB-viewer; transmembrane alpha helices named TMα_1_–α_14_ shown in dark gray and beta sheets in yellow. Conserved amino acids within the sequences are highlighted in black. Substituted residues among river and seawater dolphins are indicated in black.
(DOC)

S2 Fig Scheme of the mitochondrial electron transport chain and ND2 antiporter model. Upper figure: Simplified drawing of the mammalian electron transport chain with the five complexes that are involved in oxidative phosphorylation over the mitochondria. Lower figure: Proposed antiporter like model of ND2. Left: ND2 model for freshwater species. Right: ND2 model for saltwater species.
(TIF)

S1 Table Accession numbers for sequences produced in this study and additional sequences used for phylogenetic analyses. (XLSX)

S2 Table Parameters of codon models tested in HyPhy. The model names match those of Table 2.
(XLSX)
