# Supplementary material for: Initial Evidence for Adaptive Selection on the NADH Subunit Two of Freshwater Dolphins by Analyses of Mitochondrial Genomes
Source: PLoS One. 2015 May 6;10(5):e0123543. doi: 10.1371/journal.pone.0123543 (PMC4422622; doi:10.1371/journal.pone.0123543)
Supplement: S1 Fig — Alignment of the S. guianensis and S. fluviatilis predicted amino acid sequence with E. coli NuoN (PDB: 3RKO:D). Secondary structural representation of the predicted dolphin NADH dehydrogenase (EC 1.6.99.3) subunit 2 motifs from the PDB-viewer; transmembrane alpha helices named TMα1–α14 shown in dark gray and beta sheets in yellow. Conserved amino acids within the sequences are highlighted in black. Substituted residues among river and seawater dolphins are indicated in black. (DOC) [file pone.0123543.s001.doc]

S1 Figure

MprD_B_Sub MNNFVILPILIPLLSAILLI-FMTKNL-MLMRIFSTAASAIGIVISGILVQTVFGIL

E_Coli MTITPQNLIALLPLLIVGLTVVVVMLSIAWRRNHF---LNATLSVIG-LNAALVSLW-

**TMα1 TMα2**

MprD_B_Sub LVQTVFTKGIQTLSLGGWKAPYGIVLAADQ**FA**SLLVLTTAIIGLLVGLYSFRS-VGE-KR

E_Coli FVQVSLWFVGQ--AGAMDVTPLMRV---DG**FA**MLYTGLVLLASLATCTFAYPWLEGYNDN

H_sapiens ----------------------------------------------------------MN

S_fluviatilis ----------------------------------------------------------MN

S_guianensis ----------------------------------------------------------MN

**TMα3**

MprD_B_Sub ERSFYYSGVQFLLAGVSGAFLTGDLFNMYVFF**E**LLLIASYMLI-VLGGTKIQLRESL**KY**I

E_Coli KDEFYLLVLIAALGGIL-LANANHLASLFLGI**E**LISLPLFGLVGYAFRQKRSLEASI**KY**T H_sapiens PLAQPVIYSTIFAGTLI-TALSSHWFFTWVGL**E**MNMLAFIPVL-TKKMNPRSTEAAI**KY**F S_fluviatilis PFVSI**I**LLTTLILSTMI-VTISSHWLFAWIGL**E**MNMMAIIPIM-MKKPNPRATEAST**KY**F S_guianensis PFVSI**T**LLTTLILSTMI-VTISSHWLFAWIGL**E**MNMMAIIPIM-MKKPNPRATEAST**KY**F

**TMα4/TMα1 TMα5**/**TMα2**

MprD_B_Sub VFNIVSSALFVIGVGFLYAVTGTLNMADLSVKISESGQTGLITV-IGVLLLLVFGM**K**GGI

E_Coli ILSAAA**S**SFLLFGMALVYAQSGDLSFVALGKNLGDGMLNEPLLLAGFGLMIVGLGF**K**LSL H_sapiens LTQATA**S**MILLMAILFNNMLSGQWTMTNTTNQY------------SSLMIMMAMAM**K**LGM S_fluviatilis LTQATA**S**SLLMLAIIINLMHSSQWTIMKLFDPT------------ASILMTMALAI**K**LGL S_guianensis LTQATA**S**SLLMLAIIINLMHSSQWTIMKLFDPT------------ASILMTMALAI**K**LGL

**TMα6/TMα3 TMα7a/TMα4a**

MprD_B_Sub F**P**LYF**W**L**P**GSYYAP**P**AAISALFGALLTKVGLYAITRVFTLIFIHDTAFTHQLMIWLAALT

E_Coli V**P**FHL**W**T**P**DVYQGA**P**APVSTFLATASKIAIFGVVMRLFLYAPVGDSEAIRVVLAIIAFAS H_sapiens A**P**FHF**W**V**P**EVTQGT**P**LTSGLLLLTWQKLAPISIMYQI---SP---SLNV-SLLLTLSILS S_fluviatilis S**P**FHF**W**V**P**EVTQGI**P**LSTGLILLTWQKLAP**V**SILYQI---SP---SINL-HLMITMS**L**LS S_guianensis S**P**FHF**W**V**P**EVTQGI**P**LSTGLILLTWQKLAP**I**SILYQI---SP---SINL-HLMITMS**F**LS

**TMα7b/TMα4b TMα8**/**TMα5**  **TMα9/TMα6**

MprD_B_Sub VIF**G**VIGS**L**AYSNVMKIVI**Y**NI**I**TAV**G**VILFGVAVHTPASIQGAIYYLIHDMLIKGALFM

E_Coli IIF**G**NLMA**L**SQTNIKRLLG**Y**SS**I**SHL**G**YLLVALIALQT----GEMSMEAVGVYLAGYLFS H_sapiens IMA**G**SWGG**L**NQTQLRKILA**Y**SS**I**THM**G**WMMAVLPYNPN------MTILNLTI----YIIL S_fluviatilis ILI**G**GWGG**L**NQTQLRKIMA**Y**SS**I**AHM**G**WMTAILPYNPT------FTLLNLLI----YIVM S_guianensis ILI**G**GWGG**L**NQTQLRKIMA**Y**SS**I**AHM**G**WMTAILPYNPT------FTLLNLLI----YIVM

**TMα10/TMα7 TMα11/TMα8**

MprD_B_Sub LAGTLIA---------LTGTASLHKMGGLIKRYPVLGWMFFISAI**S**LA**G**I**P**PLS**GF**VG**K**F

E_Coli SLGAFGVVSLMSSPYRGPDADSLFSYRGLFWHRPILAAVMTVMML**S**LA**G**I**P**MTL**GF**IG**K**F H_sapiens TTTAFLLLNLNSS-------TTTLLLSRTWNKLTWLTPLIPSTLL**S**LG**G**L**P**PLT**GF**LP**K**W S_fluviatilis TFTMFMLLIQNST-------TTTLLLSQMWNTTPIMTTFTMLTLL**S**MG**G**L**P**PLT**GF**MP**K**W S_guianensis TFTMFMLLIQNST-------TTTLLLSQMWNTTPIMTTFTMLTLL**S**MG**G**L**P**PLT**GF**MP**K**W

**TMα12a/TMα9a TMα12b/**

MprD_B_Sub KIAEGGFAEGEFTISMLILLSSLLV**LY**SVL**R**IFIHAFWGEEKETPKPNHRTA-KGLLYPA

E_Coli YVLAVGVQAHLWWLVGAVVVGSAIG**LY**YYL**R**VAVSLYLHAPE---QPGRDAPSNWQYSAG H_sapiens AIIEEFTKNNSLIIPTIMATITLLN**LY**FYL**R**LIYSTSITLLP----MSNNVKMKWQFEHT S_fluviatilis MIIQELTKNDTLILPTLMAITALLN**LY**FYM**R**L**A**YSTTLTLFP----SSNNMKMKWQFYPT S_guianensis MIIQELTKNDTLILPTLMAITALLN**LY**FYM**R**L**T**YSTTLTLFP----SSNNMKMKWQFYPT

**TMα9b TMα13/TMα10**

MprD_B_Sub AIFLLLSLLFG**L**G-TEWVSPYVDQAAET**L**LNPEKYIEAVLKE

E_Coli GIVVLISALLV**L**VLGVWPQPLISIV--R**L**AMPLM--------

H_sapiens KPTPFLPTLIA**L**TTLLLPISPFMLM--I**L**-------------

S_fluviatilis KQMTLLPTAIV**L**STMLLPLTPT**F**FI--L**L**-------------

S_guianensis KQMTLLPTAIV**L**STMLLPLTPT**L**FI--L**L**-------------

**TMα14/TMα11**
