# Supplementary figures and images for: Initial Evidence for Adaptive Selection on the NADH Subunit Two of Freshwater Dolphins by Analyses of Mitochondrial Genomes
Source: PLoS One. 2015 May 6;10(5):e0123543. doi: 10.1371/journal.pone.0123543 (PMC4422622; doi:10.1371/journal.pone.0123543)

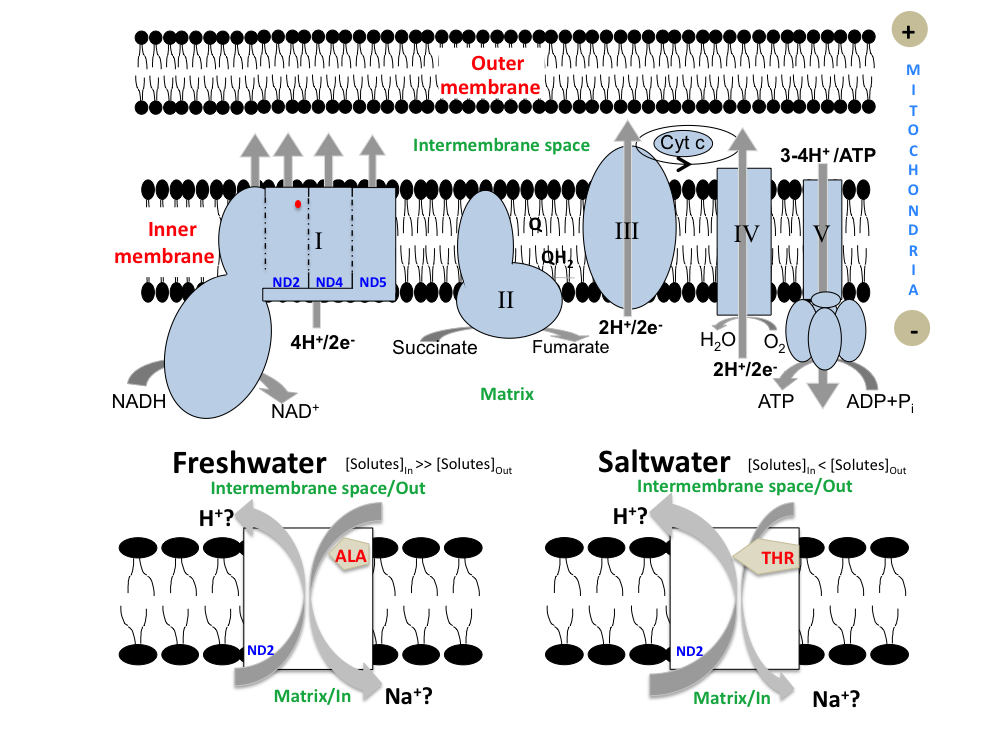

Supplement: S2 Fig — Upper figure: Simplified drawing of the mammalian electron transport chain with the five complexes that are involved in oxidative phosphorylation over the mitochondria. Lower figure: Proposed antiporter like model of ND2. Left: ND2 model for freshwater species. Right: ND2 model for saltwater species. (TIF) [file pone.0123543.s002.tif]
